# Hirayama’s disease associated with cervical deformity and spinal cord compression: a case report from Sweden

**DOI:** 10.1007/s00701-024-05982-7

**Published:** 2024-02-10

**Authors:** Johan Wänman, Per Anders Persson, Lukas Bobinski

**Affiliations:** 1 Department of Surgical and Perioperative Sciences, Umeå University, Orthopaedics, Umeå, Sweden; 2 Division of Neuroradiology, Umeå University, Umeå, Sweden

**Keywords:** Hirayama’s disease, Spinal cord compression

## Abstract

**Background:**

Hirayama’s disease (HD) is most common in young males, and previous studies are predominantly from Asian countries. The cause of HD is unknown but the most common theory about the pathology speculates on forward bending that causes a compression of the dura mater and the anterior horn of the spinal cord against the vertebra during an overstretch flexion that may result in myelopathy. Both anterior and posterior cervical surgical approaches have been shown to be effective in stopping the disease and improving function; however, HD is also reported to be a self-limited disease, and treatment with a cervical collar may be an alternative for these patients.

**Case report:**

We report HD in a 17-year-old male from Sweden who underwent surgical treatment with a 2 level anterior cervical discectomy and fusion (ACDF) due to neurological progression from HD after conservative treatment.

**Conclusion:**

HD is rare and is easily overlooked. Surgical intervention shows promising results for neurological progression, but HD is also reported to be a self-limited disease.

## Introduction

Hirayama’s disease (HD) was first described by Keizo Hirayama in 1959 [1]. HD. The most common theory about the pathogenesis of HD suggests that forward bending of the cervical spine causes forward shift of the spinal cord with stretching of the vessels due to a tight dural canal. This cascade can induce distortion of the microcirculation, increased intramedullary pressure, and venous stagnation in the posterior epidural plexus with compression of anterior horns of the spinal cord [2]. Repetitive compression may result in myelopathy with atrophy of the anterior horn of the cervical spinal cord [3]. HD is rare and most previous reports are founded on young males 15–25 years of age and of Chinese or Indian descent [4–7]. To our knowledge, no cases of HD have been described in Scandinavia.

HD occurs mostly sporadic, although familial occurrences are also reported but are quite rare [8]. Treatment with a cervical collar is the first line of choice, but surgical intervention may also be considered in patients with rapid neurological deterioration that according to the literature can progress toward spastic paraparesis [6, 9–11].

Herein, we report a case of a 17-year-old male from Sweden who presented with a cervical deformity causing progressive unilateral weakness and muscle atrophy. Based on clinical presentation, electroneuromyography (ENMG), and magnetic resonance imaging (MRI), the patient was diagnosed with HD. He underwent surgical decompression, correction, and stabilisation with a 2 level anterior cervical discectomy and fusion (ACDF) and anterior release (grade 4 cervical osteotomy).

## Case presentation

**Fig. 1:**
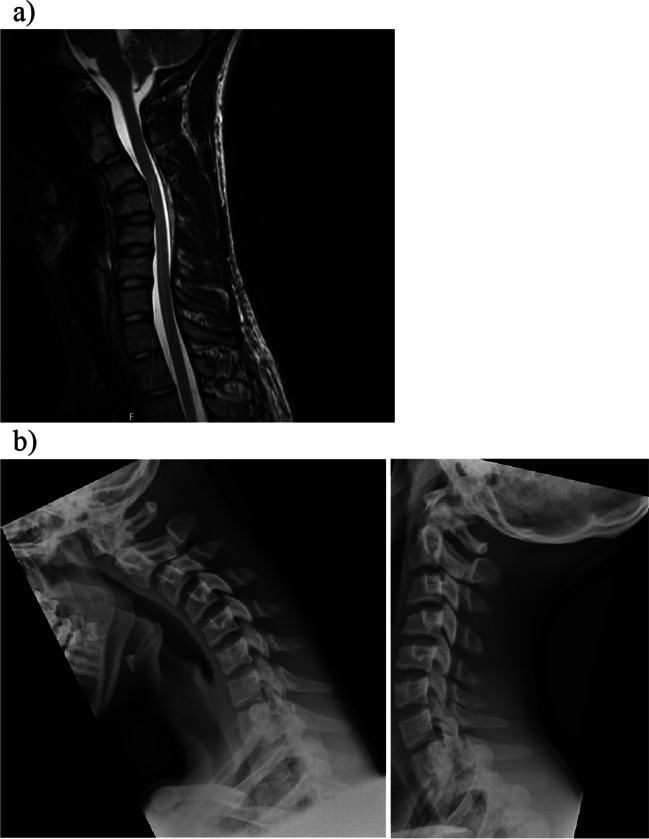
**a** Pre-operative sagittal non flexion T2-weighted MRI of Hirayama’s disease with kyphosis at C2-C5 and anterior dural shifting with epidural flow-voids. **b** Pre-operative flexion and extension x-ray revealed a kyphotic deformity between segments C3 and C5 that partially reduced in hyperextension

A 15-year-old male from Sweden with a history of hypermobility with long standing cervical pain without other relevant medical conditions was referred to our clinic. The patient complained about diffuse pain in the entire spine that was mostly concentrated in the cervical region. He described sensation of a temporary relief when he performed self-manipulation of the cervical spine under hyperflexion. He did not complain about neuropathic pain. There was no familiar history of any specific systemic diseases. He underwent genetical examination with suspicion of Ehlers-Danlos syndrome. The results were negative, and he received the diagnosis of an unspecific hypermobility syndrome. His neurological status upon the initial consultation was normal. The cranial nerve functions were normal, and there were no signs of visible atrophy or weakness in his fascial expression. The deep tendon reflexes were normal. Both deep and superficial sensory examinations, as well as the coordination, were normal. The only pathological finding was significant cervical kyphosis that was visible at the cervical radiological examination. The patient was initially referred for physiotherapy but 1 year later, he was referred back to us due to weakness in his left arm. The neurological examination at that time revealed decreased strength (4 out of 5) in corresponding C5, C6, and C7 nerve roots in his left arm with mild atrophy of the deltoid in comparison to his right arm. There were no other pathological findings in his neurological status that would indicate the development of myelopathy. Further examination with ENMG revealed moderate to severe affection of the anterior horn of the spinal cord from C5-C7 on the left side. Repeated magnetic resonance imaging (MRI) of the cervical spine showed enlargement of the posterior epidural space (Fig. 1). At the age of 17, the patient received the diagnosis of HD. The diagnosis of HD was based on a combination of clinical, electrophysiological, and radiographic findings. Flexion/extension radiological examination revealed kyphotic deformity between segments C3-C5 that partially reduced in hyperextension (Fig. 1b). Due to progression of the neurological symptoms and persistent mechanical cervical pain, we decided to treat the patient surgically. The patient and his legal guardian consented to a 2-level anterior cervical discectomy and fusion of segments C3–C5.

## Surgery

After uneventful intubation, the patient was positioned supine with the head resting in adjustable holder. Under direct control with fluoroscopy and continuous intraoperative monitoring with SSEP and MEP, the patient’s head was gradually lowered to achieve a hyperextension. This enabled partial, close reduction of the cervical kyphosis and gave better access to the cervical spine. MEP and SSEP remained unchanged throughout the reduction manoeuvre. After sterile preparation and dressing, the anterior cervical dissection was carried out, and discectomy of C3/C4 and C4/C5 was executed including resection of the posterior longitudinal ligament. The dura was completely exposed and distended, and the epidural vascular plexus was also clearly visible. To achieve further correction, resection of uncovertebral joints in segment C3/C4 was carried out. The reduction was completed by implantation of two hyperlordotic disc spacers (7° each) largest height diameters and footprint at C3/C4 and C4/C5 (Colonial® Globus Medical) positioned flush with the anterior border of the vertebra to create the lordosis and fixation with the cervical plate (Venture®, Medtronic) with six screws under direct control with fluoroscopy.

Repeated MEPs and SSEPs were unchanged in comparison to the baseline. The wound was then meticulously washed with saline solution and closed in layers with water-tight sutures. The skin was closed with absorbable sutures. In accordance with our local routine, an active deep wound drainage was left in place for 24 h.

**Fig. 2:**
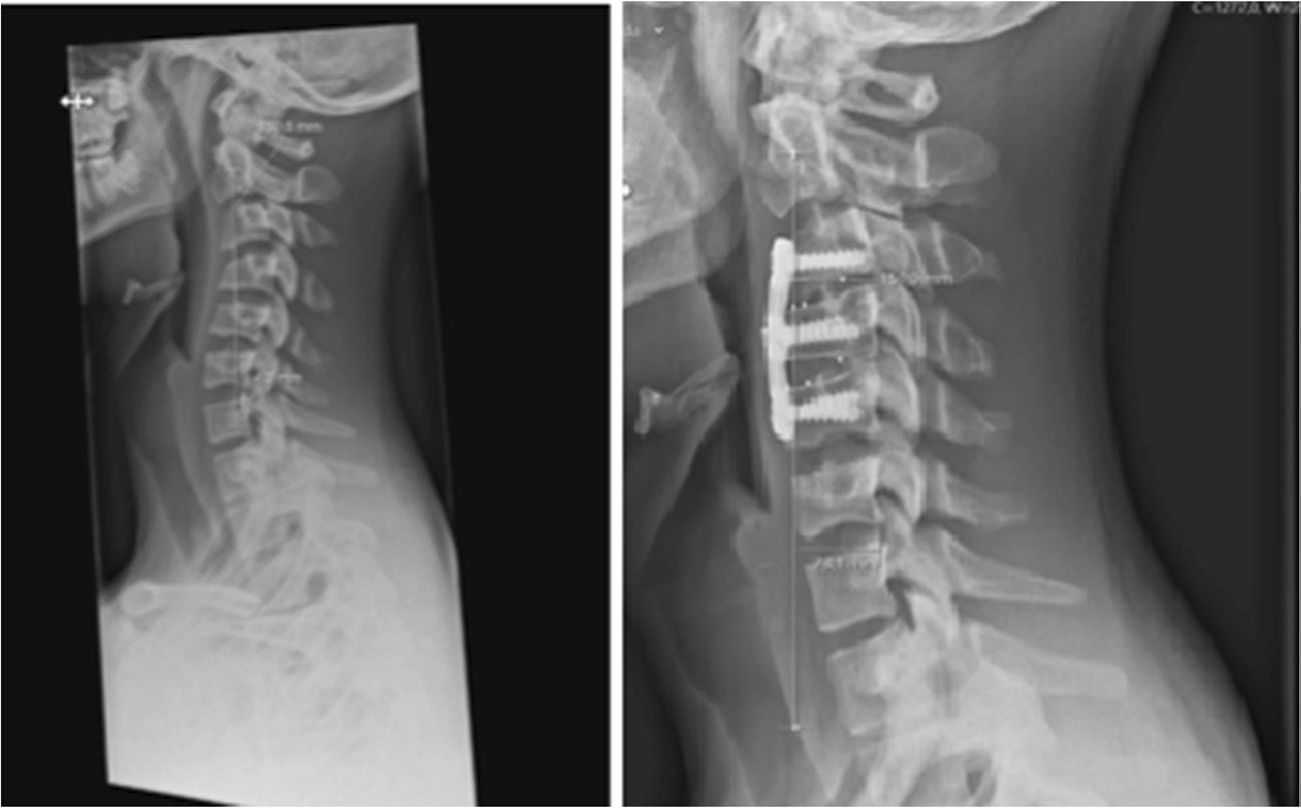
Pre and postoperative X-ray after surgical treatment with anterior cervical discectomy and fusion C3–C5. The cCVA before surgery was 4.3 mm and 23.1 mm after surgery. There was a clear reduction in the Ishihara index at the latest follow-up but with changes in alignment in the subaxial spine leading to changes in the cSVA (from 3 to 27 mm) (Table 1)

The post-operative period was uneventful. Pain control, swallowing, and articulation were normal. Cervical pain improved, but the patient complained about subjective stiffness. The neurological status remained unchanged. The post-operative radiological examination demonstrated clear improvement of cervical lordosis as well as the Ishihara index. The C2 sagittal vertical axis (cSVA) and T1 slope minus cervical lordosis indicated sufficient correction of the cervical kyphosis (Fig. 2 and Table 1).

**Table 1:** C2–C7 values in kyphosis, C3–C5 values in kyphosis, C5–C7 values in lordosis, TS-CL, T1 slope, and C2 slope. One-year post-op, the same measurements showed further improvement with C3 6.1 mm, C4 6.5 mm, and C5 4.8 mm

	Before surgery	1-year post-op follow-up	2-year post-op follow-up	3-year post-op follow-up
cSVA	3 mm	16 mm	16 mm	27 mm
C2–C7	13°	10°	9°	5°
C3–C5	30°	13°	13°	13°
C5–C7	21°	8°	7°	13°
TS-CL	9.8°	12.2°	14°	18°
T1 slope	23°	23°	23°	23°
C2 slope	15°	17°	22°	22°

## Follow-up

**Fig. 3:**
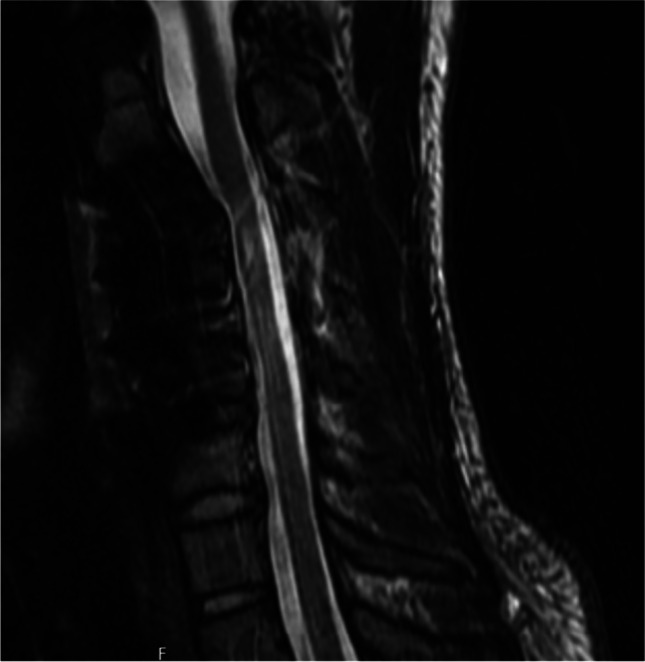
Post-op MR nearly 3 years after surgery demonstrated development of myelomalacia at the level of C4 (apex of cervical kyphosis)

Just 1 week post-operatively, the patient sought our outpatient clinic because of further decreased strength in the deltoids and biceps on the left side. Upon examination, he presented with a muscular strength corresponding to 3 out of 5 in the deltoideus and 3 + out of 5 in the biceps. An emergency MRI (Fig. 3) demonstrated reduced cervical kyphosis and an increased space for the spinal cord at the levels of the surgery. ENMG was also performed and had a similar pattern to that before surgery. This presented a suspicion of post-operative C5 palsy syndrome. The patient received short-term steroids and was referred for physiotherapy. At the end of the first year of follow-up, his strength significantly improved and stabilised to the baseline level before surgery. Radiological examination demonstrated solid fusion between the operated segments. The patient remained active. The difference in strength between the left and right arm was difficult to confirm objectively during examination. However, muscle atrophy of the left underarm remained unchanged, and subjectively, the patient noticed the site difference. Cervical pain improved significantly but the patient continued to complain about pain in the thoracic spine and the urge to manipulate his neck to ease neck discomfort. Radiological examination during the last follow-up (3 years after surgery) revealed significant reciprocal changes in the subaxial spine that led to deterioration in the vertical cervical alignment with cSVA extending to almost 3 cm at the last follow-up. Furthermore, control MRI demonstrated development of myelomalacia at the level of C4 (apex of cervical kyphosis) (Fig. 3). Finally, repeated ENMG showed disappearance of the anterior horn affection, but with remaining lowered amplitudes of both C4 and C5 nerve roots on the left side. Details regarding cervical parameters are listed in Table 1. At this point, we discussed with the patient about a complementary posterior correction with instrumentation between C2 and C5 but he declined due to a positive development with less pain and a stable neurological function.

## Discussion

HD is a rare disease that can easily be overlooked. Most reports of HD are from Asian countries; the present case study is to our knowledge the first case report from northern Europe with a native patient. A delay of a HD diagnose is common but should be avoided due to the progressive, deteriorative nature of the disease. The characteristic unilateral upper extremity weakness should arouse suspicion for HD. Regular supine position MRI may reveal localised spinal cord atrophy, oedema, distention of the epidural venous plexus, and an abnormal cervical curvature [8]. When HD is suspected, additional MRI of the cervical spine in the flexed position can be ordered since it is much more sensitive for demonstrating anterior spinal cord shift and flattering due to posterior compression of the congested epidural plexus [12]. A recent systematic review about HD highlighted the importance of dynamic MRI in flexion and static MRI in both the neutral position and flexion [13] but other studies have not revealed any radiological and clinical relation to neck flexion [14, 15]. Although HD is described as a self-limiting condition, increasing numbers of publications suggest that HD carries the risk for progression to spastic paraparesis [6]. Hence, the decision about surgical treatment should be carefully discussed with the patient and limited to severe cases with a rapid neurological progression [16]. Both anterior and posterior surgical procedures have been proposed but anterior surgery with ACDF is most commonly reported in the literature [10, 17, 18]. The results of surgery are promising with a more than an 80% chance of clinical improvement, with no significant difference between the surgical approaches [18]. The main difference, in our case, was that the patient presented at first consultation with a severely painful cervical sigmoidal deformity but with normal neurological examinations. Even though there were no changes in MEPs and SSEPs during the surgery and complete anterior release and decompression of the spinal cord and exiting bilateral C4 and C5 nerve roots, the patient developed left-sided C5 palsy. The reason for the C5 root deficit is difficult to explain, C5 palsy after anterior approach is very rare but has been descried in the literature [19]. There were no changes in MEP and SSEP stimulation through-out the surgery that could explain the new onset of weakness corresponding with C5 nerve root occurred 1 week after surgery. Furthermore, the deficit disappeared spontaneously after short administration of per-oral steroid, which supports our diagnosis.

However, during the follow-up, we observed spontaneous reciprocal changes triggered by C3-C5 correction that led to deterioration in cSVA. The general alignment measured as the Ishihara index and cSVA remained within acceptable limits during follow-up. We can only speculate about whether the C2/C3 segments were involved in the deformity and therefore should also be corrected and stabilised. The high C2 slope, responsible for elevated values of cSVA, might suggest this. However, the risk and benefit ratios need to be carefully considered, especially when excessive correction of the cervical spine is required. The control cervical MR revealed successful decompression of the spinal cord and regression of vascular congestion but with development of myelomalacia (Fig. 3). The indication for surgery in HD can be debated, but the conservative treatment is challenged since surgical treatment by either anterior or posterior cervical approaches has demonstrated to be effective in stopping the disease and improving the function of affected limbs [20].

## Conclusions

HD is rare and easily overlooked. Most case studies are from Asian countries. Surgical intervention shows promising results for neurological progression, but HD is also reported to be a self-limited disease.

## Data Availability

The datasets used and/or analysed during the current study are available from the corresponding author on reasonable request.

## Code availability

The datasets used and/or analysed during the current study are available from the corresponding author on reasonable request.
